# Asymmetric Supercapacitor Based on Biomass-Derived Carbon Electrodes Functionalized with NdFeB

**DOI:** 10.3390/ma19061257

**Published:** 2026-03-22

**Authors:** Ahmad Reshad Delawary, Constantin Bubulinca, Natalia E. Kazantseva, Petr Saha, Quoc Bao Le, Ram K. Gupta, Rudolf Kiefer

**Affiliations:** 1 University Institute, Tomas Bata University in Zlin, Nad Ovčírnou 3685, 760 01 Zlin, Czech Republic; delawary@utb.cz (A.R.D.); bubulinca@utb.cz (C.B.); nekazan@yahoo.com (N.E.K.); saha@utb.cz (P.S.); 2 National Institute for Materials Advancement, Pittsburg State University, Pittsburg, KS 66762, USA; qle@pittstate.edu (Q.B.L.); rgupta@pittstate.edu (R.K.G.); 3 Department of Chemistry, Pittsburg State University, 1701 S. Broadway Street, Pittsburg, KS 66762, USA; 4 Conducting Polymers in Composites and Applications Research Group, Faculty of Applied Sciences, Ton Duc Thang University, Ho Chi Minh City 700000, Vietnam

**Keywords:** active carbon, supercapacitor, NdFeB, PANI, biomass

## Abstract

Supercapacitors (SCs) are highly attractive energy storage devices, and modern research is focused on using waste materials to reduce environmental impact. This study processed biowaste from local brewery production to produce a highly specific mesoporous activated carbon (AC) for SC electrode scaffolds. Polyaniline (PANI) was synthesized and incorporated into the AC scaffold, thereby enhancing performance. The AC and PANI combination (**ACP**) achieved a specific capacitance of 173.7 F/g at 1 A/g, with 92% retention after 5000 cycles. Using NdFeB (**ACN**) particles, the anode showed a specific capacitance of 127 F/g and over 99% retention. An asymmetrical **ACN//ACP** cell demonstrated promising performance with 70% efficiency. This study highlights the potential of using biowaste for high-performance SC electrodes and the effective synergy between AC and PANI.

## 1. Introduction

The consumption of fossil fuels leads to environmental issues such as air pollution and energy shortages, underscoring the urgent need for efficient energy storage devices. Electrochemical devices, such as batteries and supercapacitors, offer practical solutions for efficient energy storage and distribution [1,2]. Supercapacitors (SCs), also known as electrochemical capacitors, have attracted significant attention due to their unique features. These include high power density, rapid charging and discharging capabilities, a remarkably long lifespan exceeding 100,000 cycles, and superior electrochemical stability compared to batteries. This device is classified by mechanism into electric-double-layer capacitors (EDLCs), pseudocapacitors, and hybrid capacitors [3,4]. EDLC operates on the electrostatic principle, in which energy is stored when an electrical potential is applied, enabling the absorption of ions from the electrolyte onto the surface of porous carbon [5,6].

However, SCs face low energy-density challenges, limiting their use in the transportation sector. To improve SCs’ energy density [7], researchers focus on developing advanced electrode materials, including transition metals, carbon-based materials [8], conductive polymers [9], and their composites [10]. Carbon-based materials, such as activated carbons (ACs), are particularly suitable for electrical double-layer capacitors (EDLCs) due to their high surface area, chemical stability, and electronic conductivity [11]. Due to its cost-effectiveness and ecological harmlessness, biomass from agricultural waste can be a valuable resource, offering a suitable and sustainable source for generating ACs [12]. Particularly in supercapacitor applications, the growing need for sustainable materials with exceptional electrochemical performance has recently spurred research on biomass-derived carbon and transition-metal composites [6]. On the other hand, these carbonaceous materials, such as ACs, suffer various limitations, including low energy density in EDLCs. Researchers are investigating enhanced carbon materials such as graphene, carbon nanotubes, and heteroatom-doped carbons, as well as hybridizing activated carbon with pseudocapacitive materials, including conducting polymers and metal oxides, to tackle these issues.

Due to their unique electrical characteristics and structural benefits, conductive polymers have gained significant importance in the fabrication of supercapacitors [13,14,15]. SCs are energy storage devices characterized by long cycle life, rapid charge/discharge performance, and high power density, bridging the gap between traditional capacitors and batteries. Various conducting polymers are utilized in supercapacitors, including Polyaniline (PANI), Polypyrrole (PPY), and Poly (3,4-ethylenedioxythiophene) (PEDOT) [16]. Among them, PANI has attracted special interest among electrochemical researchers since it is a promising electrode material for SCs, known for its excellent electrical conductivity and high specific capacitance. However, PANI faces challenges with cycle stability, impacting its practical application. Despite these challenges, PANI’s superior electrical conductivity and high specific capacitance have led to its widespread investigation [17]. Aziz et al. reported the synthesis of electrochemically deposited polyaniline on Tomato-leaf-derived hierarchical carbon for an SC. The fabricated asymmetric supercapacitor exhibits 95% capacitance retention after 10,000 cycles. Neodymium–iron–boron (NdFeB) is also a potential material for energy storage devices [18]. NdFeB, which contains rare-earth elements, has shown promising performance as a magnet in Li-ion batteries and SC systems. However, significant amounts of NdFeB are wasted as dust during production.

The novelty lies in utilizing Brewer’s spent grain (BSG)-derived carbon in two-step carbonizations and in using NdFeB powder for SC electrodes. PANI/AC and AC/NdFeB composites were prepared via co-assembly. The electrochemical properties of the obtained materials were studied to assess their suitability as electrode materials for asymmetric supercapacitors.

Recently, the conversion of bio-waste into scopes that meet industrial demands for energy device manufacturing has become increasingly vital to minimize the environmental hazard crisis. To the best of our knowledge, no prior research has been conducted or reported on this topic, making this work a novel contribution to the field. BSG-derived AC/PANI and AC/NdFeB were initially utilized for the asymmetric supercapacitor. The results highlight the potential of using biowaste and NdFeB for high-performance SC electrodes, demonstrating that AC from BSG and NdFeB powder can significantly enhance SC energy storage performance. This approach not only leverages sustainable materials but also addresses material waste, offering a promising pathway for future research and development in sustainable energy storage technologies.

## 2. Materials and Methods

### 2.1. Materials

Potassium nitrate (KNO_3_, 99%) was purchased from Sigma-Aldrich (Taufkirchen, Germany). Neodymium–iron–boron nanoparticles/nanopowder (NdFeB, 99.9%, APS < 100 nm) were obtained from Nano Research Elements (Southall, UK). All solvents were sourced from local suppliers, PENTA (Praha, Czech Republic) and VWR Chemicals (Stříbrná Skalice, Czech Republic), and they were used without further purification.

Brewer’s spent grain (BSG) waste was collected from a local brewery in Zlín, Czech Republic. To prevent microbial contamination, the biowaste was oven-dried at 110 °C for 48 h. The dried precursor was then micronized using a ball-milling machine (LMLW-320/2, Roermond, The Netherlands) and sieved to obtain micro-sized particles.

### 2.2. Hydrothermal Carbonization

The 5 g of micronized BSG was mixed with 60 mL of distilled water to obtain a homogenous solution. The mixture was transferred into an 80 mL stainless-steel autoclave and then thermally treated in the reactor at 180 °C for 3 h. After cooling to room temperature, the obtained precursor was rinsed with deionized water until the filtrate was clear, removing the dissolved impurities. The residue was dried in an oven at 110 °C for 24 h, and the resulting hydrochar was designated HT-180 °C.

### 2.3. Preparation of Activated BSG Carbon

The activation and pyrolysis processes were carried out according to the literature process [19,20,21]. HT-180 (2 g) was mixed with KOH (5 g) in a 1:2.5 ratio using a magnetic stirrer (neo LabLine, Heidelberg, Germany to achieve a homogeneous solution in 100 mL of deionized water. The prepared solution was then poured into a large Petri dish and dried in an oven at 110 °C. Once dried, the material was weighed and transferred to a GSL-1600X tubular furnace (Carbolite Gero, Neuhausen, Germany), where it was carbonized at 800 °C for 10 h under an Ar flow, with a heating rate of 5 °C min^−1^. After carbonization, the furnace was allowed to cool to room temperature. The resulting BSG carbonized material was then ball-milled to obtain micro-sized particles. The material was subsequently washed with 3% HCl and distilled water until the pH reached approximately 7. Finally, the sample was dried at 105 °C for 12 h, and the final product was labeled AC. The AC preparation process is illustrated in Scheme S1.

### 2.4. PANI Preparation

The process was followed in our previous publication [22]. Aniline (1.86 g, 0.02 mol) was dissolved in 50 mL of 0.2 M HCl and transferred to a 200 mL beaker. While stirring, 50 mL of ammonium persulfate (APS) solution (5.7 g, 0.025 mol in 0.2 M HCl) was gradually added. The beaker was then covered, and the reaction mixture was stirred at room temperature for 24 h. The resulting PANI precipitate was filtered and washed thoroughly with ethanol and deionized water. The obtained precipitate was dispersed in 150 mL of ethanol and ultrasonicated for 12 min. After filtration, the precipitate was redispersed in another 150 mL of ethanol and ultrasonicated for an additional 30 min. The final product was stored in a refrigerator until further use.

### 2.5. Fabrication of Electrodes

A slurry was prepared by mixing AC-HT-180 (90 mg) with a polytetrafluoroethylene (PTFE) solution (10 wt% of AC-HT-180) in 1 mL of ethanol. The resulting slurry was coated into circular shapes (radius = 0.25 cm) onto NiO mesh and subsequently compressed. The electrode fabricated solely from AC-HT-180 was designated as AC.

A similar slurry preparation was followed for composite electrodes, incorporating either PANI (20 wt%) after drying at 60 °C or NdFeB (20 wt%) into the mixture. The resulting slurries were coated and compressed onto NiO meshes. The electrode composed of AC-HT-180 and PANI was named **ACP**, while the electrode containing AC-HT-180 and NdFeB was designated **ACN**.

### 2.6. Characterization

The chemical composition of the samples was analyzed by ATR-FTIR spectroscopy using a Nicolet iS10 (Thermo Scientific, Waltham, MA, USA) equipped with an ATR sampling accessory and a Ge crystal plate. The specific surface areas were determined using the Brunauer–Emmett–Teller (BET) method, while nitrogen adsorption–desorption measurements were carried out with a Micromeritics 2020 volumetric adsorption analyzer. Before analysis, all samples were heat-pretreated under vacuum at 70 °C for 3 h. The pore structures were examined using a NANOSEM 450 (FEI, Hillsboro, OR, USA) scanning electron microscope (SEM) operated at 5 kV under 90 Pa.

The electrochemical performance of the prepared electrodes was evaluated at room temperature using cyclic voltammetry (CV), galvanostatic charge–discharge (GCD) tests, and electrochemical impedance spectroscopy (EIS) with an Autolab PGSTAT-128N potentiostat (Methrom AG, Herisau, Switzerland). These measurements were conducted in a three-electrode configuration, consisting of the working electrode, a large-surface-area platinum wire counter electrode, and an Ag/AgCl reference electrode in a 1 M KNO_3_ aqueous solution. For two-electrode system testing, identical electrodes were assembled in a Swagelok cell, separated by an NKK-MPF30AC supercapacitor separator (ANR Technologies, Shenzhen, Guangdong, China). The cell was filled with electrolytes and sealed with parafilm for long-term retention studies.

## 3. Results and Discussion

This research utilized biological waste from a local brewing company in Zlín province, Czechia, to synthesize AC for SC. BSG was first carbonized and then activated to produce activated carbon, which significantly altered its chemical and structural characteristics. The preparation of AC was displayed in Scheme S1. Using biowaste for recycling demonstrates an eco-friendly approach and addresses environmental impact. During carbonization, the main lignocellulosic components of BSG, cellulose, hemicellulose, and lignin, undergo thermal decomposition under limited oxygen conditions. This process removes volatile compounds, such as water, carbon dioxide, and other organic gases, leaving a carbon-rich solid with an initial porous structure. As a result, the carbonized BSG becomes more thermally stable and forms an aromatic carbon framework, which serves as the backbone for subsequent activation [20].

After carbonization, the resulting char contains increased fixed carbon content, reduced oxygen and hydrogen content, and a partially developed pore structure. The decomposition of cellulose and hemicellulose during heating creates micropores, while lignin contributes to the formation of a stable carbon matrix due to its aromatic structure and higher carbon yield. Additionally, some residual oxygen-containing functional groups remain on the carbon surface. They can enhance surface reactivity during activation. The natural fibrous morphology inherited from the original plant structure is also partially preserved, contributing to the formation of interconnected pore channels within the carbonized material [23].

Following carbonization, activation further develops the porosity and increases the surface area of the carbonized BSG, producing AC. During activation, reactions occur between the activating agent and the carbon matrix. These reactions etch the carbon structure, widen existing pores, and create new micropores and mesopores. Consequently, the AC derived from carbonized BSG exhibits significantly higher surface area, greater pore volume, and a hierarchical pore structure. Furthermore, it consists of micropores for charge storage and mesopores for electrolyte ion transport [24]. These characteristics make the BSG-derived AC particularly suitable for SC applications, where high surface area and efficient ion diffusion are essential for improving electrochemical performance.

Using biowaste for recycling demonstrates an eco-friendly approach and addresses environmental impact. AC, with its high specific surface area, enhances SC performance by increasing its electrical double-layer capacitance (EDLC). However, AC often exhibits pseudocapacitance, which can be challenging to account for. This study introduced PANI and NdFeB metal complexes to overcome this. This combination improved the pseudocapacitance coefficient and increased the material’s capacitance in negative and positive operational environments. This innovative approach enhances SC performance and efficiency, contributing to sustainable energy storage solutions.

In this study, we investigated the synergy between NdFeB, AC, and PANI in composite structures for supercapacitor electrodes. Various electrolytes are commonly used in supercapacitor applications, including acidic (H_2_SO_4_), alkaline (KOH), and neutral (KNO_3_) solutions. When conducting polymers such as PANI are incorporated into electrode structures, acidic and neutral electrolytes are generally preferred due to their favorable interactions with the material’s surface [16]. In addition to PANI, we used NdFeB in our electrode fabricated on Ni foam and selected a neutral 1 M KNO_3_ electrolyte to minimize potential chemical side reactions during operation.

However, it should be noted that BSG is used as the supply for this study due to its convenience in collection. Several other lignocellulosic or protein-/mineral-rich wastes can mimic BSG’s ability to yield high-surface-area, mesoporous carbons for supercapacitors when carbonized and KOH-activated [25]. Nut shells, such as walnut or macadamia, have high lignin and fixed-carbon contents as well as very rigid shell architectures. Hence, they withstand aggressive KOH activation and develop hierarchical micro/mesoporous structures comparable to those of BSG-derived carbons [26]. Agricultural residues such as sugarcane bagasse, corn cobs, and palm fibers possess vascular plant structures and a mixed cellulose–hemicellulose–lignin composition. These compounds can translate into interconnected mesopores and macropores after carbonization and activation [27]. They provide ion-transport pathways analogous to those from BSG’s fibrous husk morphology. In practice, any waste that combines moderate-to-high lignin, some heteroatom content, and a naturally hierarchical plant microstructure can serve as a viable alternative to BSG for producing mesoporous carbons tailored for SC applications [28].

### 3.1. XRD and FT-IR Studies

**Figure 1:**
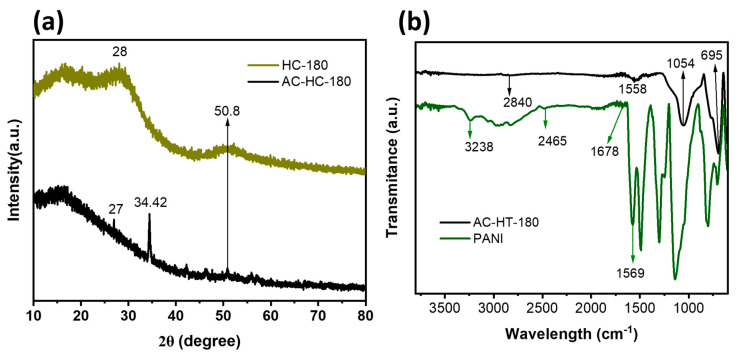
(**a**) The XRD pattern of HT-180 and AC-HT-180; (**b**) the FTIR of AC-HT-180 and PANI.

The XRD patterns of the biomass after the hydrothermal treatment exhibit prominent peaks at approximately 28° and 50.8° (Figure 1a), corresponding to the C(002) and C(100) planes of amorphous carbon, respectively. These peaks indicate the presence of condensed aromatic carbon structures formed during the treatment process [29]. These structural characteristics are largely preserved during KOH activation, where potassium-containing compounds (such as K_2_CO_3_ and K_2_O) form and intercalate into the carbon framework. Upon subsequent washing with HCl, these compounds dissolve, leaving behind a highly porous activated carbon with an enlarged surface area [29]. The appearance of sharper peaks suggests a higher degree of aromatic ring condensation within the activated carbon structure [30]. After activation, the peak of amorphous carbon C(002) shifts to 27° and an additional peak appears at 34.42°, which is likely associated with the (311) crystallographic plane [31].

The FTIR spectra of AC and synthesized PANI are shown in Figure 1b. The peak at 1558 cm^−1^ is assigned to the C=O stretching vibration of carboxylic acid groups, while the small band at 2840 cm^−1^ is associated with CH_2_ stretching vibrations. The absorption band at 1054 cm^−1^ corresponds to C–O–C bonding, and the peak at 695 cm^−1^ is attributed to C=O bending vibrations [32]. For PANI, the FTIR spectrum displays a characteristic peak at 3238 cm^−1^, which corresponds to the N–H stretching vibration of the amino groups. The peak at 2465 cm^−1^ is attributed to N–H vibrations of unsaturated amine groups. The band at 1678 cm^−1^ is assigned to carbonyl (C=O) stretching vibration. Additionally, the band at 1569 cm^−1^ corresponds to C=C stretching of the quinoid ring, a key indicator of a higher-oxidized state, confirming the successful formation of polyaniline [33].

### 3.2. Morphologies Study and Specific Area Analysis

**Figure 2:**
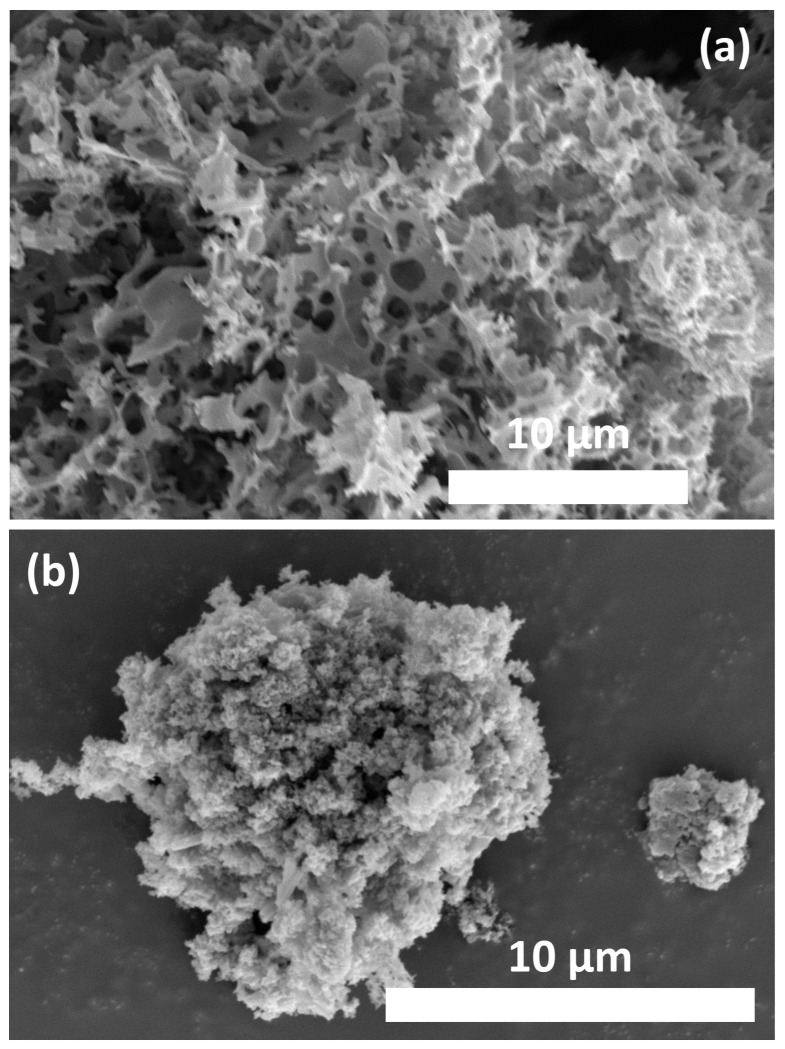
The SEM images of (**a**) AC-HT-180 and (**b**) PANI.

Figure 2 displays SEM images of activated carbon derived from brewer’s biowaste. The sample, prepared with a pre-carbonized carbon-to-KOH ratio of 1:2.5 and pyrolyzed at 800 °C for 2 h, exhibits a rough, porous surface. This porosity results from gasification during activation, forming K_2_O, CO, CO_2_, K_2_CO_3_, and K, along with H_2_ and H_2_O [34]. At temperatures above 700 °C, K_2_O and K_2_CO_3_ form, etching the carbon skeleton via redox reactions. At 800 °C, metallic potassium (K) diffuses into the carbon structure, enhancing porosity. Post-reaction rinsing with HCl dissolves K and its compounds, producing a highly porous activated carbon structure. The mesoporous nature of AC-HT-180 suggests a large specific surface area.

The structure of PANI as nanoparticles is also shown in Figure 2b. The formation of PANI with this morphology can be attributed to stirring during polymerization, which leads to strong interactions between conjugated polymer chains. This structure can be readily dispersed into the AC structure.

### 3.3. Specific Surface Area and Pore Size Analysis

**Figure 3:**
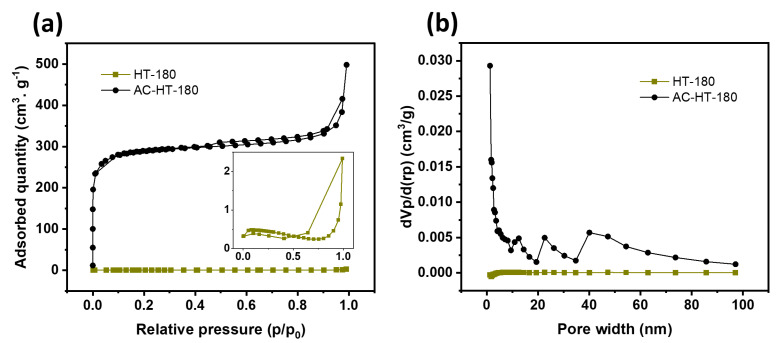
Adsorption/desorption isotherm (**a**) and pore size distribution (**b**) of HT-180 and AC-HT-180 d.

Figure 3a presents nitrogen adsorption/desorption isotherms for carbonized BSG (HT-180) and activated carbon (AC-HT-180). AC-HT-180 displays a type II isotherm, indicating monolayer and multilayer adsorption with a weak hysteresis loop due to capillary condensation. In contrast, HT-180 exhibits a type III isotherm with a larger hysteresis loop at relative pressures above p/p0 = 0.6, suggesting capillary condensation in a mesoporous structure. AC-HT-180 shows superior nitrogen adsorption due to a significantly larger specific surface area (1081 m^2^/g vs. 1.8 m^2^/g for HT-180). HT-180′s desorption curve drops sharply at p/p0 = 0.9, stabilizing around p/p0 = 0.6 due to the liquid-to-gas nitrogen transition.

Figure 3b shows pore size distribution based on BJH analysis. HT-180 displays a range of micro, macro, and mesopores with a 2–20 nm concentration. AC-HT-180’s pores are mainly in the meso- and macroporous regions, with an average size under 5 nm and peaks at 12, 23, and 41 nm. The BET isotherm indicates a specific surface area of 1081 m^2^/g, consistent with morphological results. High surface-to-volume ratios and abundant mesopores in materials enhance charge storage and transfer kinetics, which are essential for advanced energy storage [35].

In SC electrodes, micropores (<2 nm) primarily contribute to charge storage by providing a large surface area for the formation of the electric double layer, thereby enhancing capacitance. These nanoconfined spaces allow for high-density charge storage, with pores smaller than 1 nm specifically enabling increased capacitance through partial desolvation of electrolyte ions, even when the ions are larger than the pore diameter. In contrast, mesopores (2–50 nm) facilitate the diffusion of electrolyte ions and reduce ion transport resistance, enabling faster charge–discharge processes. Therefore, an optimal hierarchical pore structure combining micro- and mesopores is crucial, as micropores provide abundant active sites for charge accumulation. The mesopores serve as ion-buffering reservoirs and transport pathways, thereby improving the electrode material’s overall electrochemical performance [36].

### 3.4. Electrochemical Properties of the 3-Electrode System

We compressed the composite slurry on NiO foam to understand the electrochemical performance of the electrodes in a three-electrode system using 1 M KNO_3_ as the electrolyte. The NdFeB-containing electrode exhibited a stable performance as an anode, operating within a negative potential window. In contrast, the AC and PANI (ACP) electrodes demonstrated efficient capacitive behavior in a positive window.

**Figure 4:**
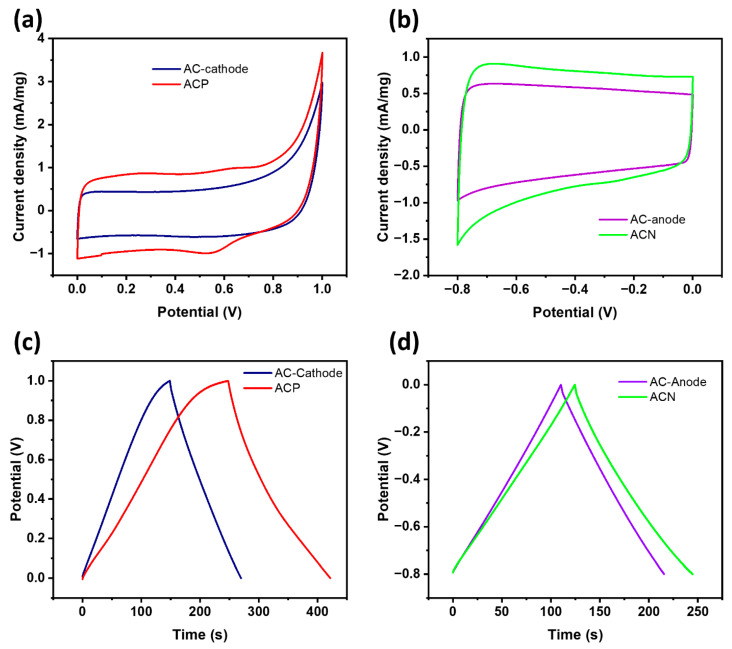
Three-electrode system performance of cyclic voltammetry at 5 mV/s of (**a**) AC and **ACP** in positive potential windows and (**b**) AC and **ACN** in negative potential windows; charge–discharge plots at 1 A/g of (**c**) AC-Cathode, **ACP**, and (**d**) AC-Anode and **ACN**.

Figure 4 presents the electrochemical properties of these electrodes, tested with Pt and Ag/AgCl reference electrodes. The **ACN** electrode exhibited optimal performance in the −0.8 to 0 V range, whereas the **ACP** electrode performed best between 0 and 1 V. The electrochemical EDLC behavior of carbon-based materials was evident in their quasi-rectangular CV curves (Figure 4a). These curves indicate efficient ion adsorption and desorption at the electrode interface, confirming the high EDLC performance of AC materials [37]. Additionally, the CV profiles of AC as an anode maintained an ideal double-layer capacitance even at higher scan rates (Figure S1) [38,39]. Similarly, Figure 4b shows that AC and ACN electrodes maintain high quasi-rectangular CV shapes at a 100 mV/s scan rate, demonstrating their excellent double-layer capacitive behavior.

The enhanced capacitance of ACP and ACN electrodes relative to pristine AC is attributed to the synergistic effects of PANI and NdFeB. The presence of PANI introduced faradaic redox reactions, as evidenced by distinct redox peaks in **ACP**’s CV curve [40]. These peaks correspond to the reversible insertion and desorption of NO_3_^−^ ions in PANI, as described by Equation (1) [41]:

(PANI)_n_ + ny NO_3_^−^ → [PANI^y+^ NO_3_^−^] + ny e^−^ (1)

The doping degree, denoted by ‘y,’ represents the ratio of charges within the polymer to the number of monomer units [42]. PANI also exhibits favorable conductivity characteristics. As it operates, PANI readily shifts between oxidation states, including leucoemeraldine (fully reduced), emeraldine (partially oxidized), and pernigraniline (fully oxidized) when subjected to an applied current [43]. ACP’s spectrum displays redox peaks associated with its performance in acidic aqueous electrolytes, indicating a transition towards PANI’s fully oxidized pernigraniline state (at 0.65/0.53 V) [22]. Theoretically, the transition of PANI would partially oxidize to emeraldine before becoming the pernigraniline state. However, in the CV curves of ACP, the redox peak can be found at approximately 0.3 V, while the anodic peak is difficult to identify due to the coverage effect of active carbon within the composite.

The BET results further support the electrochemical findings. AC-HT-180 exhibited a significantly larger specific surface area (1081 m^2^/g) than carbonized BSG (1.8 m^2^/g). This high surface area, combined with its mesoporous structure, provides abundant active sites for ion adsorption, which is crucial for improving charge storage and transfer kinetics. The pore-size distribution analysis (Figure 3b) revealed that AC-HT-180 contained a dominant concentration of mesopores (<5 nm) with additional peaks at 12, 23, and 41 nm, facilitating efficient diffusion of electrolyte ions. The presence of mesopores and macropores in ACN and ACP electrodes promotes rapid ion transport, reducing diffusion resistance and enhancing capacitive performance.

The galvanostatic charge–discharge (GCD) curves provided a clear insight into the specific capacitance of electrode materials. As depicted in Figure 4b,c, all composites displayed triangular shapes when tested under current densities of 1 A/g, indicating excellent electrochemical reversibility [1]. The specific capacitance Cs was determined (Equation (2)).

(2) $$C_{s}=\frac{I\cdot \Delta t}{m\cdot \;\Delta V}$$

where *I* represents the applied current (A), Δ*t* (s) stands for the discharge time, *m* (g) denotes the mass of the active electrode material, and ΔV (V) signifies the discharge potential [44].

The GCD curves indicate that incorporating **PANI** and the **NdFeB** composite extends the discharge time, resulting in a higher specific capacitance than pristine AC. This enhancement is attributed to improved ion diffusion in the electrolyte during charge/discharge, which facilitates the formation of double electric layers at the electrode surface. This study presents the GCD curves of the anode in a widely adopted convention in electrochemical energy storage research. This representation is commonly used because it directly reflects the practical operation of devices. Numerous supercapacitor studies follow this standard method to ensure consistency in performance evaluation and comparison [45,46,47,48]. Therefore, this approach provides an accurate and meaningful analysis of the electrochemical behavior of the studied materials.

The nearly triangular shape of **ACN**’s GCD curve highlights its strong EDLC properties, characterized by a linear relationship between potential and time [49]. Furthermore, the absence of peaks in the CV curves suggests minimal Faradaic reactions during scanning. A detailed analysis of GCD curves at varying scan rates is provided in Figure S2 and Table S1. However, at a closer glance, we can see a slight change that makes them less linear. This phenomenon occurs because of NdFeB’s contribution to the structure. During GCD, different components oxidize at different potentials. During discharge, a passivation film of iron or neodymium hydroxides may form on the surface. This film increases resistance over time, causing the voltage to drop non-linearly [50].

**Figure 5:**
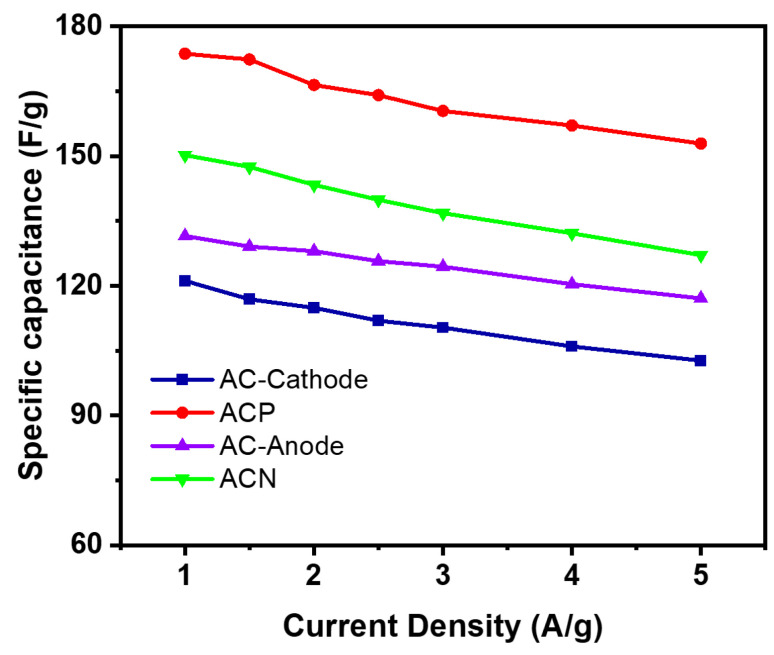
Specific capacitance of electrode made of **AC, ACP,** and **ACN** calculated from GCD curves.

The observed reduction in capacitance at higher current densities for electrodes made from these composites is attributed to ion reactions with electrode materials during scanning (Figure 5). Elevated scanning rates hinder the accessibility of electrolyte ions to electrode surfaces. It is established that electrode capacitance correlates with structural configurations and morphological characteristics. The porous structure of the samples facilitates ionic conductivity, while composite components significantly enhance ion transport within the electrodes [22].

The specific capacitance of **ACN** was 150.21 F/g at 1 A/g, while the **AC** anode was 131.51 F/g. With increasing scan rate, specific capacitance decreased, reaching 127.10 F/g for **ACN** and 117.07 F/g for **AC** at 5 A/g. This reduction correlates with faster sweep rates, which shorten ion–electrode interaction time, explaining the decline in specific capacitance. For **ACP** and **AC**-cathode, specific capacitance decreased from 173.7 F/g and 121.15 F/g at 1 A/g to 152.89 F/g and 102.68 F/g at 5 A/g, respectively. These findings highlight the influence of sweep rate variations on charge/discharge efficacy, shedding light on ion–electrode interactions under different conditions.

### 3.5. Working Performance of Symmetrical SCs

**Figure 6:**
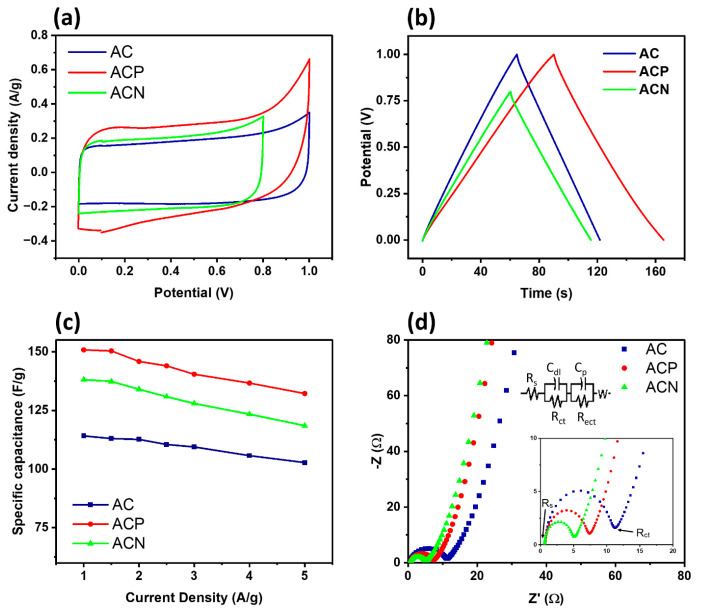
Two-electrode symmetrical SC system performance of (**a**) cyclic voltammetry at 5 mV/s, (**b**) charge–discharge plots at 1 A/g, (**c**) specific capacitance calculated from GCD curves, and (**d**) electrical impedance with the equivalent circuit.

Supercapacitor cells with **AC**, **ACN**, and **ACP** electrodes in 1 M KNO3 aqueous solution were studied by CV, GCD, and EIS over positive potentials. CV curves for **AC** and **ACP** spanned 0–1 V, while ACN covered 0–0.8 V. Figure 6 and Figure S3 show CV curves for various potential scan rates (5–100 mV), revealing favorable capacitive behavior in all samples. Despite increasing scan rates, CV curves maintain their shape, indicating substantial EDLC in the materials. A comparative analysis shows higher specific capacitance for ACP and ACN compared to AC at the same scan rate.

The GCD curves of the composites, evaluated at different scan rates, exhibit a relatively symmetrical, linear triangular shape (Figure 6b). These curves demonstrate the cells’ commendable electrochemical capacitive behavior. The specific capacitance (Cs) of the two-electrode SC was determined through calculation [51] given in Equation (3).

(3) $$C_{s}^{\prime }=2\frac{I\cdot ∆t}{m\cdot ∆V}$$

Figure S3 and Table S2 illustrate specific capacitance details for two-electrode supercapacitor (SC) cells, mirroring trends in three-electrode systems. The notably high specific capacitance of the cells highlights material stability. While SC electrode application often compromises performance, our materials maintain stability and reliable capacitance. **ACP** exhibits superior performance due to PANI’s pseudocapacitance properties, with capacitance of 150.8 F/g at 1 A/g and 132.2 F/g at 5 A/g (Figure 6c). Other samples show satisfactory results (138.11 F/g for **ACN**, 114 F/g for **AC**), with minor reductions at higher scan rates. Despite these declines, overall performance remains appropriate, affirming the potential applicability and versatility of our electrode materials in supercapacitors.

We also conducted the EIS tests on symmetric SCs to analyze ion transport mechanisms and retention capabilities. Data collected over a 0.01 Hz to 100 kHz frequency range at 0.01 V revealed Nyquist plots with semicircles in the high- and mid-frequency ranges, indicative of charge-transfer resistance (R_ct_) and interfacial ion-diffusion behavior [52]. The R_ct_ values derived from semicircle diameters reflect differences in capacitive behavior and ion-transport efficiency at electrode–electrolyte interfaces [53]. Among the samples, **ACP** exhibited the lowest R_ct_ value (4.5 Ω), indicating superior capacitive behavior and robust retention. The **ACN** cell displayed an R_ct_ of 6.8 Ω, slightly higher than ACP, indicating potentially long-term retention but slightly increased ion transport resistance, which may lead to lower retention than **ACN**. In contrast, the **AC** electrode showed the highest Rct (10.66 Ω), indicating a greater charge-transfer barrier, which correlates with reduced retention due to inefficient ion movement at the interface [54].

To further interpret ion transport resistance, an equivalent circuit model was incorporated in Figure 6d, consisting of R_s_ (series resistance), R_ct_ (charge transfer resistance), C_p_ (double-layer capacitance), and W (Warburg impedance) for ion diffusion. The linear region in the low-frequency range corresponds to the Warburg impedance, and the slope of the oblique line indicates the electrolyte ion-diffusion efficiency. A more vertical trend suggests a lower diffusion resistance, confirming improved ion mobility within composite electrodes. The near-linear behavior of **ACP** and ACN in the low-frequency region suggests reduced ion transport limitations, whereas the pronounced deviation in AC indicates hindered ion diffusion [53]. These findings emphasize the role of composite structures in enhancing charge storage efficiency and reducing interfacial resistance, making **ACP** and **ACN** more promising for high-performance SC applications retention.

**Figure 7:**
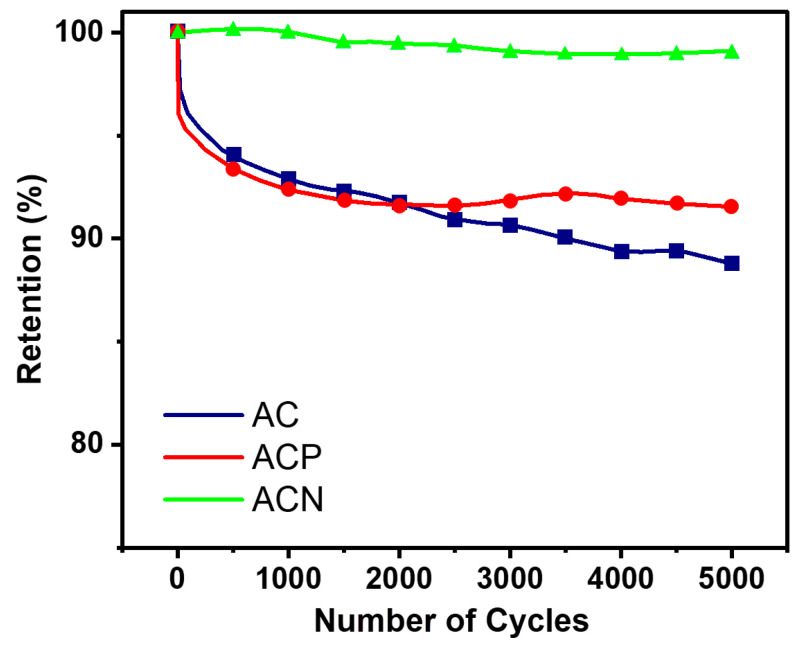
The cyclic capacity retention during 5000 cycles at 3 A/g of symmetrical cell of **AC**, **ACP** and **ACN**.

Figure 7 presents the cycling stability of two-electrode symmetrical SCs for 5000 cycles at a current density of 3 A/g for all samples. As expected from the EIS data, the AC-based cell, which primarily relies on a non-Faradaic electric EDLC mechanism, exhibited the lowest charge–discharge retention, maintaining approximately 89% of its initial capacitance after 5000 cycles. This decline is attributed to the gradual loss of accessible surface area for ion adsorption, a characteristic of EDLC-based materials. In contrast, the **ACN** cell demonstrated good stability, retaining over 99% of its capacitance. This high retention confirms its dominant non-Faradaic EDLC behavior, where charge storage occurs via reversible ion adsorption at the electrode–electrolyte interface. Since no significant redox reactions occur, material degradation is minimized, leading to superior cycling performance [40].

A slight increase in capacitance during the early cycles of **ACN** is attributed to gradual electrochemical activation and wetting of the composite electrode. During the initial cycling, the **ACN** electrode likely opens previously inaccessible pores in the carbon and improves electrolyte penetration into the AC/NdFeB composite. Therefore, more of the real surface area becomes active over time, as is commonly observed in porous carbon electrodes [55]. In parallel, repeated polarization can slightly modify surface functional groups and the interface at the AC/NdFeB interface. The formation of thin oxide/hydroxide layers on the Fe-rich surface creates additional electroactive sites and lowers charge-transfer resistance, resulting in a modest rise in apparent capacitance over the first hundreds of cycles. Furthermore, the small mechanical rearrangements of the hard NdFeB particles within the softer carbon/binder matrix during the first cycles can densify local percolation pathways. It can improve charge transport within existing micropores rather than creating new porosity, as reflected in a modest increase in capacitance before the electrode structure stabilizes [56].

The **ACP** electrode incorporating PANI follows a Faradaic charge-storage mechanism due to the polymer’s redox activity. It exhibited a slight decline in retention to approximately 92%, primarily due to the inherent instability of PANI’s redox-active polymer chains during prolonged cycling. The charge storage in PANI is affected by Faradaic processes, where reversible redox reactions between PANI and NO_3_^−^ ions lead to the formation of [PANI^+^ NO_3_^−^] complexes. During continuous cycling, the expansion and contraction of the polymer chains weaken the matrix, leading to structural degradation and reduced capacitance retention [41].

Meanwhile, the AC/NdFeB-based electrode exhibited a non-Faradaic charge-storage mechanism, maintaining remarkable cycling stability with over 99% retention after 5000 cycles. The NdFeB component enhances charge transfer and ion diffusion, working in concert with the porous **AC** matrix to facilitate EDLC-based ion adsorption. This balanced interaction prevents excessive structural degradation and enhances overall electrode performance. The strong chemical resistance and favorable electrode–electrolyte interactions observed in this composite make it a promising candidate for long-term energy storage applications.

### 3.6. Electrochemical Properties of Asymmetric SCs

This study’s asymmetrical supercapacitors (SCs) were meticulously constructed using **ACN**, **AC**, and **ACP** electrodes. These materials underwent rigorous testing within a specified working potential range. To ensure consistency, electrodes with an equal mass of material were used under symmetrical SC conditions with a 1 M KNO_3_ aqueous electrolyte. The working potential ranged from 0 to 1.8 V to accommodate differences between the anode and cathode of the SCs. Cells were designated based on electrode combinations and configurations. For instance, **ACN** served as the anode and **AC** as the cathode in the **ACN//AC** configuration. This comprehensive approach, incorporating novel materials such as ACN, enhances our understanding of electrode component interactions and their impact on performance across diverse electrochemical systems. The potential window of the asymmetric cell is determined based on the distinct operating performance of the anode and cathode electrodes. In separate tests, the cathode electrode exhibited optimal performance over 0–1 V, while the anode performed best between −0.8 and 0 V. In a symmetric configuration, the working window aligned well with the individual electrode performance. Therefore, when combined within an asymmetric cell, the overall working potential is the absolute sum of the cathode and anode operating potentials.

**Figure 8:**
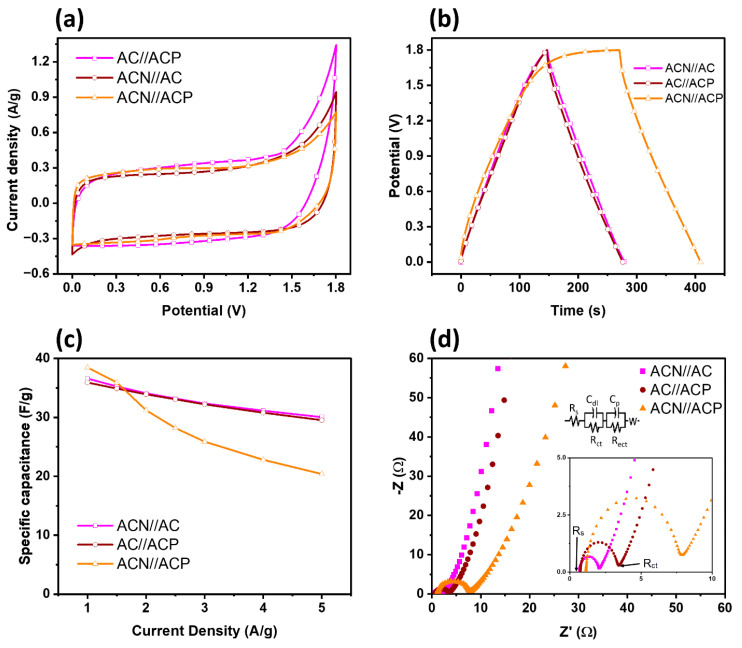
Two-electrode asymmetrical SC system performance of (**a**) cyclic voltammetry at 5 mV/s, (**b**) charge–discharge plots at 1 A/g, (**c**) specific capacitance calculated from GCD curves, and (**d**) electrical impedance and the equivalent circuit.

Figure 8 and Figure S4 show CV curves for two-electrode asymmetrical cells over potential scan rates from 5 to 100 mV. Unlike symmetrical cells, only **AC**//**ACN** CV curves deviate from a quasi-rectangular shape, indicating favorable capacitive behavior. Others show a shift indicative of pseudocapacitance when the **ACP** cathode is used. The consistent CV curve shapes with increasing scan rates highlight substantial EDLC, as shown in Figure S4 and Table S3.

Galvanostatic charge–discharge curves exhibit symmetrical and linear triangular shapes across various scan rates (Figure 8b), indicating commendable electrochemical capacitive behavior. Current density calculations were based on a single supercapacitor electrode (SC) with similar masses for both electrodes, halving the acquired data accordingly [51]. The specific capacitance (C’as) of asymmetric two-electrode SCs was calculated according to Equation (4) [51], expressed as follows:

(4) $$C_{as}^{\prime }=\frac{1}{2}\frac{I\cdot \Delta t}{m\cdot \;\Delta V}$$

Figure 8c and Figure S4 present the variation in specific capacitance of asymmetric two-electrode SC cells derived from GCD measurements at different current densities. At a current density of 1 A/g, the **ACN//ACP** cell has the highest specific capacitance (38.46 F/g), followed by **ACN//AC** (36.58 F/g) and **AC//ACP** (35.93 F/g), indicating enhanced charge storage capability due to the synergistic contribution of the electrode materials. However, as the current density increases, the specific capacitance of all cells gradually decreases due to limited ion diffusion in the electrolyte and reduced accessibility of active sites at higher charge–discharge rates [57]. Notably, the **ACN//ACP** cell showed a significant decline in capacitance, dropping to approximately 20 F/g at 5 A/g, suggesting poorer rate capability and possible interfacial resistance or ion-transport limitations. In contrast, **ACN//AC** and **AC//ACP** exhibit comparatively stable capacitance retention, maintaining values around 30 F/g at 5 A/g, indicating better electrochemical kinetics and rate performance. These results suggest that while the **ACN//ACP** cell provides higher capacitance at low current density, the **ACN//AC** and **AC//ACP** cells exhibit superior stability and rate capability.

EIS data from asymmetric SCs provide valuable insights into electrolyte–electrode interactions, particularly in the ACN//ACP cell configuration. Figure 8d highlights variations in R_ct_ across different cell configurations, revealing critical differences in charge-transfer resistance that directly influence performance. The ACN//ACP cell exhibits a higher R_ct_ of 6.73 Ω, suggesting increased resistance at the electrolyte–electrode interface. This elevated resistance may arise from hindered ion mobility within the composite structure, slowing charge transfer kinetics and potentially reducing power output. The increased **R_ct_** could also indicate a less efficient Faradaic charge-storage process at the **ACP** electrode due to PANI’s structural expansion and contraction during cycling, which can degrade interfacial conductivity over time. It also explained why the asymmetrical **ACN//ACP** cell has a lower specific capacitance as the scan rate increases. The interaction between **ACN** and **ACP** materials may partially restrict electrolyte penetration or create less efficient ion transport pathways. Such structural limitations become more pronounced at high scan rates [58].

In contrast, cells incorporating AC **and** ACN **(1.34 Ω) or** ACP **(2.74 Ω)** demonstrate significantly lower R_ct_, indicating smoother ion transport and enhanced interfacial compatibility. The lower R_ct_ values in these configurations suggest more efficient ion diffusion pathways, thereby improving charge storage and faster response times during cycling. The ACN electrode, with its optimized porosity and surface chemistry, facilitates rapid ion adsorption and desorption, thereby enhancing the system’s capacitive behavior. The elevated R_ct_ in ACN//ACP could have long-term implications for device efficiency and charge retention. A higher resistance at the electrode interface may lead to increased energy dissipation as heat, impacting the SCs’ overall energy density and stability. Additionally, repeated cycling may increase R_ct_ due to gradual material degradation, particularly in the PANI-based electrode. The high resistance slows electron transfer, particularly at high current densities.

**Figure 9:**
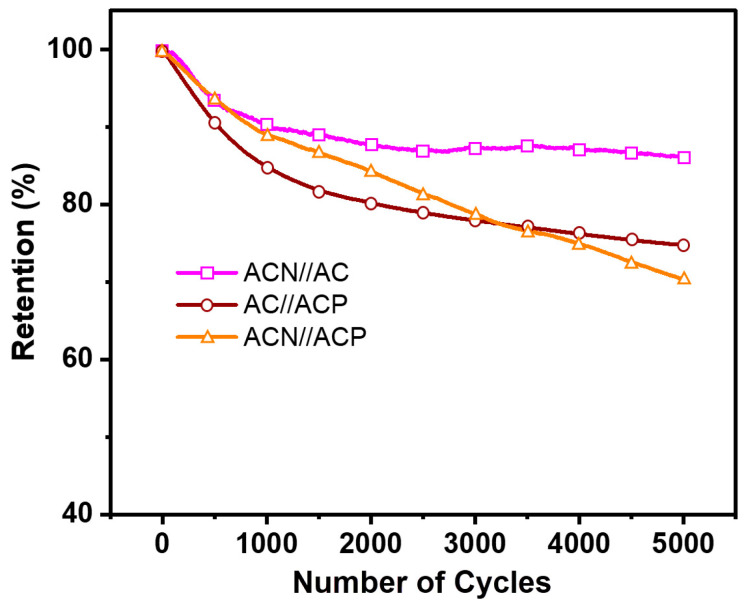
The cyclic capacity retention during 5000 cycles at 3 A/g of the asymmetrical **ACN//AC**, **AC//ACP** and **ACN//ACP** SCs.

Figure 9 displays cycling stability at a current density of 3 A/g for 5000 cycles in two-electrode asymmetrical SCs of **ACN//AC**, **AC//ACP**, and **ACN//ACP**. Retention was continuously calculated over a potential window of 0–1.8 V, similar to the CV and GCD testing conditions. Comparing retention data from symmetrical and asymmetrical SCs, electrodes made of **AC** and NdFeB are expected to exhibit higher retention due to their lower R_ct_, indicating a more favorable electrode–electrolyte interaction. After 5000 cycles, the **ACN//AC** cell retained over 86%, showcasing commendable performance despite lower retention than symmetrical SCs (99%). NdFeB electrodes also demonstrated riveting performance. Conversely, cells using PANI showed lower retention due to degradation of the conducting polymer, exacerbated by unfavorable electrode–electrolyte interactions, as indicated by the high R_ct_ from EIS [41]. Polymer chain transformation and ion interaction during cycling contribute to material degradation. However, challenges in electrolyte ion diffusion between ACN and ACP hinder long-term charge/discharge processes. Consequently, AC//ACP exhibited approximately 75% retention, while **ACN//ACP** exhibited approximately 70% retention. Despite this, exploring PANI or other conducting polymers with NdFeB and AC scaffold is possible for future investigation. In this study, PANI was added to the composite as a separate component, combined with PTFE. However, previous research showed that PANI with different modifications and conjugating approaches can yield significantly different results [22]. Therefore, scientists could decipher the conjugation method for various polymer and copolymer systems based on PANI with Ppy, PEDOT, or numerous conducting polymer composites with AC and NdFeB.

Due to the strong electrochemical performance and synergistic effects between the porous AC derived from BSG, NdFeB, and PANI, the electrochemical properties of AC/PANI (**ACP**) and AC/NdFeB (**ACN**) composite electrodes are competitive with those of other reported metal oxide-based composites (as shown in Table 1) [59,60,61,62,63,64,65,66]. Notably, research on the application of NdFeB in supercapacitors remains limited. While NdFeB has been utilized in lithium batteries [18], its role in supercapacitors has primarily been explored in the context of drive control methodologies for bonded-NdFeB-spoke-PM SM systems in city mini-buses with SCs [67]. This study demonstrates that residual NdFeB materials, which are typically discarded during production and fabrication, can be repurposed as electrode materials for long-term energy storage applications.

At a current density of 3 A/g, the **ACN//ACP** cell exhibited moderate specific capacitance in a neutral electrolyte. However, given their rate performance and cycling stability, the composite materials show potential for further improvement and long-term retention. CV analysis indicates that the **ACN** electrode primarily relies on an EDLC charge-storage mechanism, resulting in stable capacitance retention. In contrast, **ACN** experiences some capacitance decrease due to the intrinsic degradation of PANI, a limitation associated with the polymer’s redox cycling. Nevertheless, the overall cycling stability remains high, with retention exceeding 92%.

Different charge-storage mechanisms can affect retention when combining polymer-based and transition-metal composite electrodes in an asymmetric SC. However, NdFeB remains a promising material for energy storage applications, warranting further investigation to optimize its performance in supercapacitors.

## 4. Conclusions

Brewer’s spent grain from a local Zlín brewery was carbonized via a hydrothermal reaction and subsequently activated with KOH, yielding a high-mesoporous **AC** with a significant specific surface area. This **AC** served as a scaffold for SC electrode materials, alongside synthesized polyaniline (**ACP**) and NdFeB particles (**ACN**). In a three-electrode system with 1 M KNO_3_ electrolyte, the **ACN** electrode exhibited a notable capacitance of 150.21 F/g in the negative potential window, while **ACP**, as the cathode, showed a higher capacitance of 173.7 F/g. As symmetrical SCs, both materials displayed robust performance, retaining high capacitances over 5000 cycles, with **ACN** reaching over 99% and **ACP** and **AC** at around 92% and 89%, respectively. Asymmetrical SCs combining **ACN** and **ACP** with **AC** showed promising results, with retention rates exceeding 75% for **AC** with NdFeB or **PANI** individually and 70% for the combined cell after 5000 cycles. These findings highlight the potential applications of these developed electrode materials in diverse supercapacitor configurations.

## Figures and Tables

**Table 1:** Comparison of electrode working performance made of AC and metal oxides applied on SCs.

Electrode	Electrolyte	Specific Capacitance (F/g)	Current Density	Retention	Cycles	Ref
NiO@Co_3_O_4_-AC	3 M KOH	800.9	10 A/g	98.1%	5000	[59]
MnO_2_/AC	6 M KOH	398	1 A/g	62%	1000	[60]
AC/MnO_2_	0.5 M Na_2_SO_4_	745.5	5 mV/s	86%	3000	[61]
NiO//AC	2 M KOH	568.7	0.5 A/g	90.6%	5000	[62]
MgO/AC	6 M KOH	295.3	200 mV/s	93.84%	10,000	[64]
Fe_3_O_4_/CDC	1 M KOH	346.5	1 A/g	92.3%	4000	[65]
C/Co_3_O_4_	2 M KOH	54	0.1 A/g	82%	10,000	[66]
AC/PANI	1 M KNO_3_	173.7	3 A/g	92%		This work
AC/NdFeB		150.21		99%	5000	
ACP//ACN		38.46		70%		

## Data Availability

The original contributions presented in this study are included in the article/Supplementary Materials. Further inquiries can be directed to the corresponding author.

## Supplementary Materials

The following supporting information can be downloaded at https://www.mdpi.com/article/10.3390/ma19061257/s1, Scheme S1. The preparation process of AC. Figure S1: CV curves of AC-cathode/anode, ACP, and ACN using the three-electrode system with 1 M KNO_3_ electrolyte. Figure S2: Charge-discharge plots of AC-cathode/anode, ACP, and ACN using the three-electrode system. Figure S3: CV curves and Charge-discharge plots of symmetrical cells made of AC, ACP, and ACN. Figure S4: CV curves and Charge-discharge plots of asymmetrical ACN//AC, AC//ACP, and ACN//ACP cells. Table S1: Specific capacitance calculated from the three-electrode system’s GCD according to the current densities. Table S2: Specific capacitance calculated from the symmetrical cells’ GCD according to the current densities. Table S3: Specific capacitance calculated from the asymmetrical cells’ GCD according to the current densities.
