# Supplementary material for: Asymmetric Supercapacitor Based on Biomass-Derived Carbon Electrodes Functionalized with NdFeB
Source: Materials (Basel). 2026 Mar 22;19(6):1257. doi: 10.3390/ma19061257 (PMC13027709; doi:10.3390/ma19061257)
Supplement: Supplementary file 1 [file materials-19-01257-s001.zip › materials-4212137-supplementary.pdf]

materials

Supplementary

Asymmetric Supercapacitor Based on Biomass-Derived Carbon Electrodes Functionalized with NdFeB

Ahmad Reshad Delawary [1], Constantin Bubulinca [1], Natalia E. Kazantseva [1], Petr Saha [1], Quoc Bao Le [2], Ram K. Gupta [2,3] and Rudolf Kiefer [4,*]

[1] University Institute, Tomas Bata University in Zlin, Nad Ovčírnou 3685, 760 01 Zlin, Czech Republic; delawary@utb.cz (A.R.D.); bubulinca@utb.cz (C.B.); nekazan@yahoo.com (N.E.K.); saha@utb.cz (P.S.)
[2] National Institute for Materials Advancement, Pittsburg State University, Pittsburg, KS 66762, USA; qle@pittstate.edu (Q.B.L.); rgupta@pittstate.edu (R.K.G.)
[3] Department of Chemistry, Pittsburg State University, 1701 S. Broadway Street, Pittsburg, KS 66762, USA
[4] Conducting Polymers in Composites and Applications Research Group, Faculty of Applied Sciences, Ton Duc Thang University, Ho Chi Minh City 700000, Vietnam
* Correspondence: rudolf.kiefer@tdtu.edu.vn; Tel.: +84-792696724

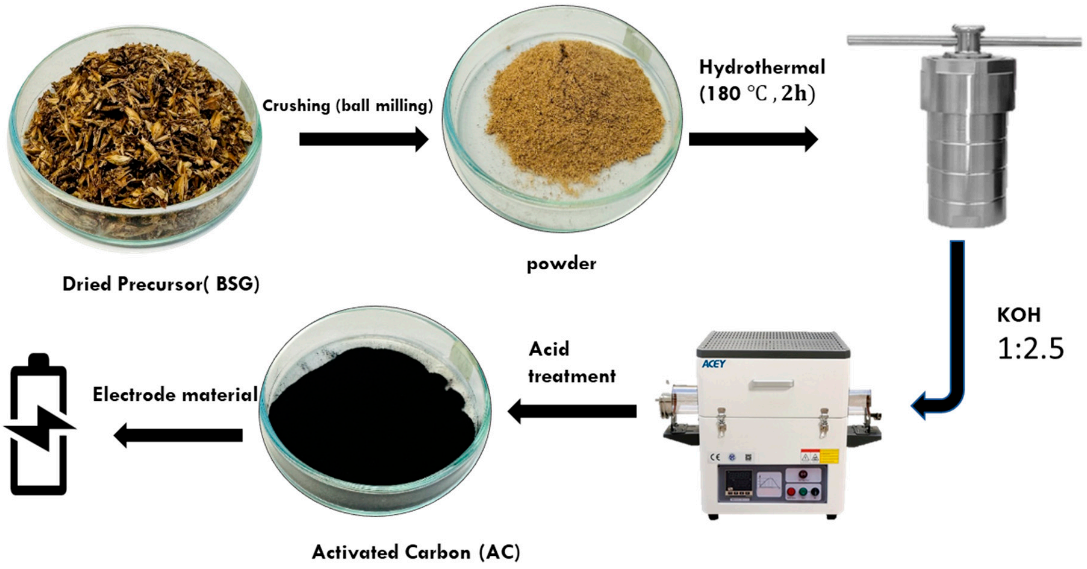

Scheme S1. The preparation process of **AC**.

 https://doi.org/10.3390/ma19061257

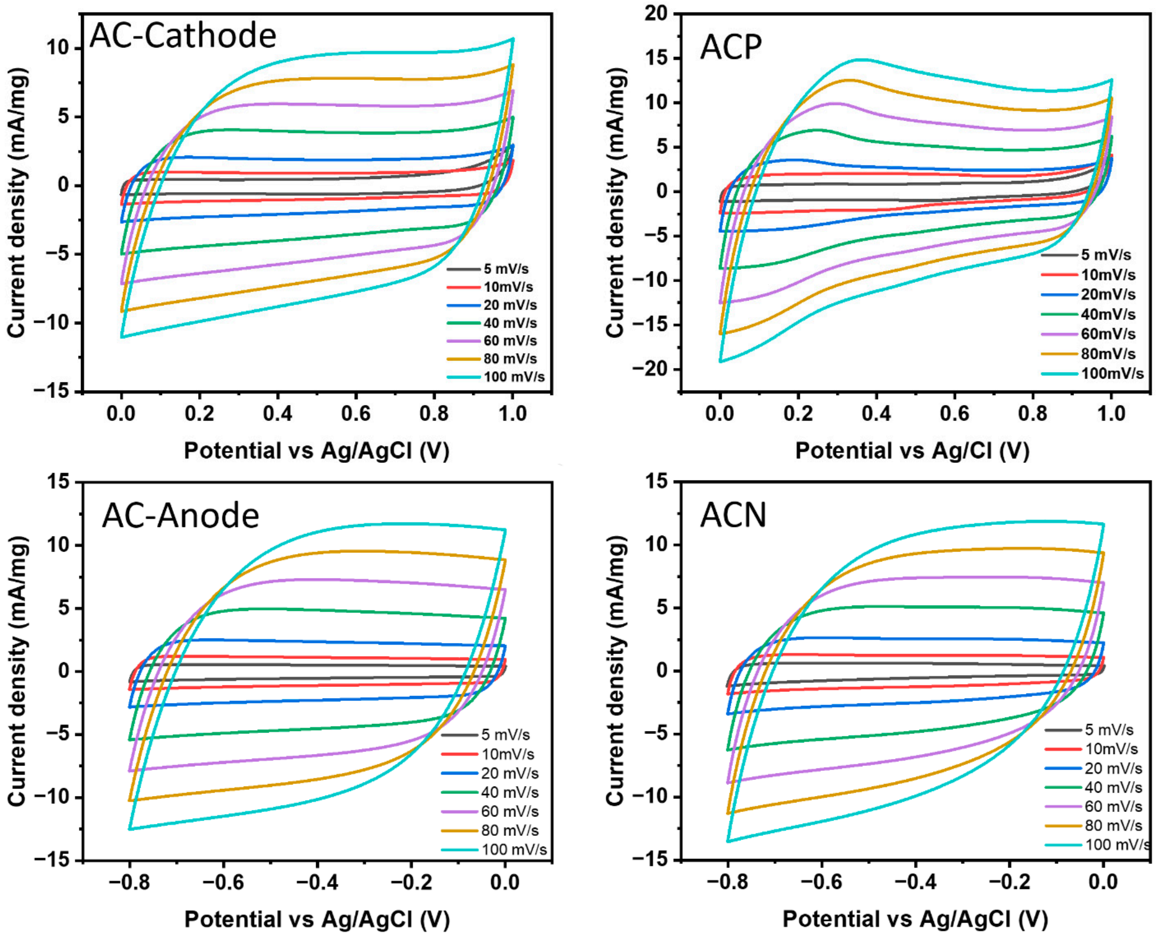

Figure S1. CV curves of AC-cathode/anode, ACP, and ACN using the three-electrode system with 1 M KNO_3 electrolyte.

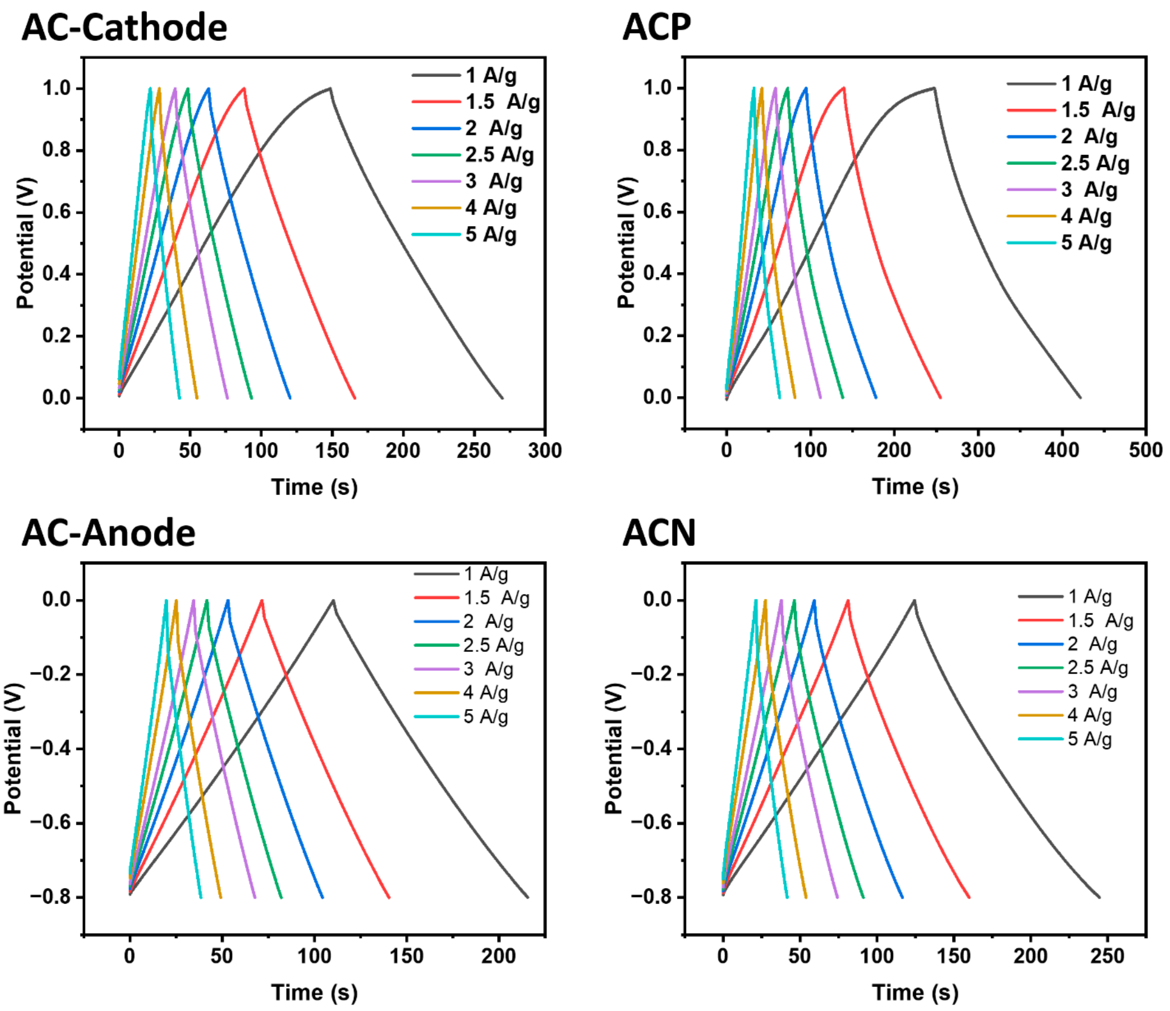

Figure S2. Charge-discharge plots of **AC-cathode/anode**, **ACP**, and **ACN** using the three-electrode system.

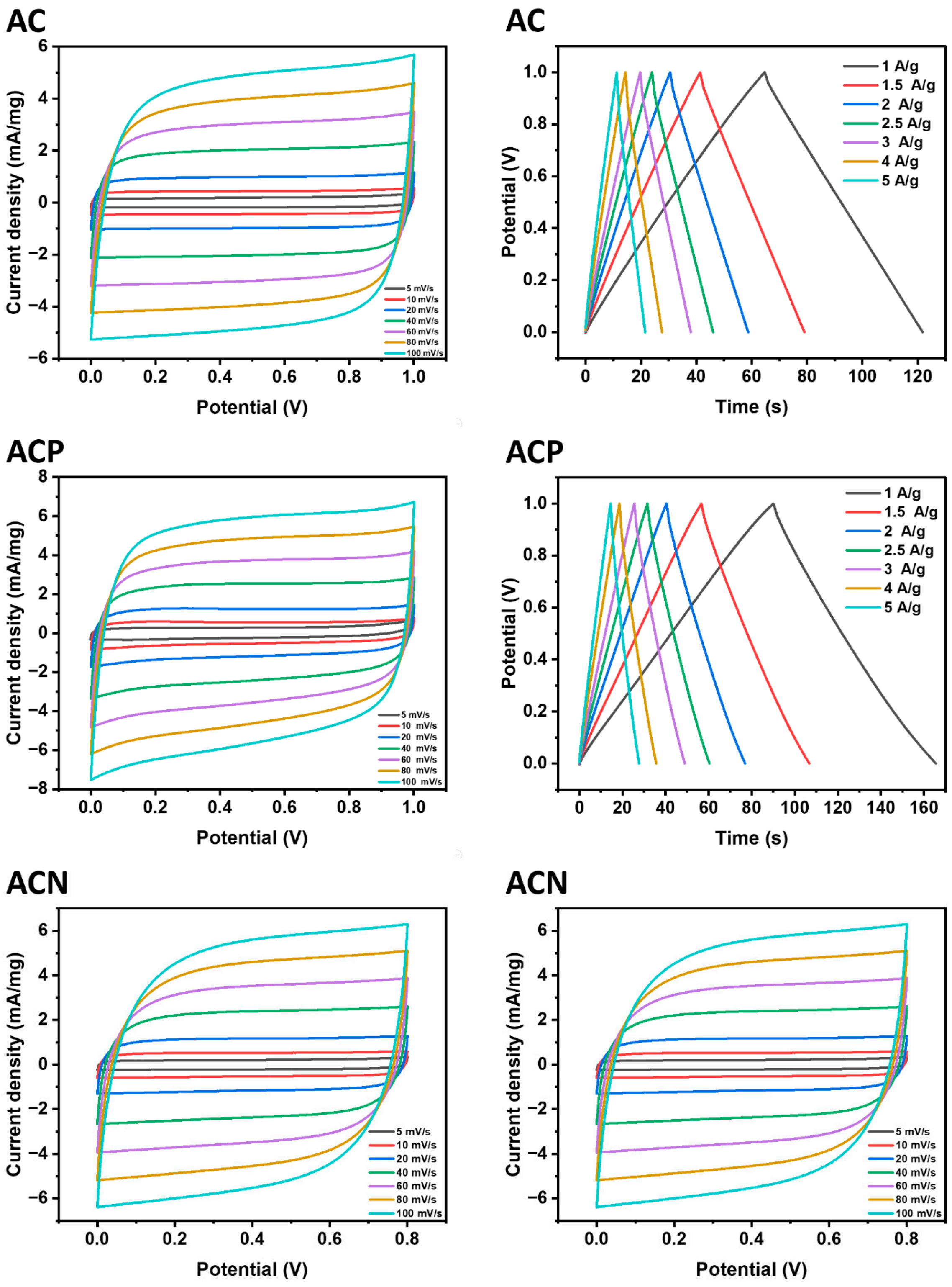

Figure S3. CV curves and Charge-discharge plots of symmetrical cells made of **AC, ACP**, and **ACN**.

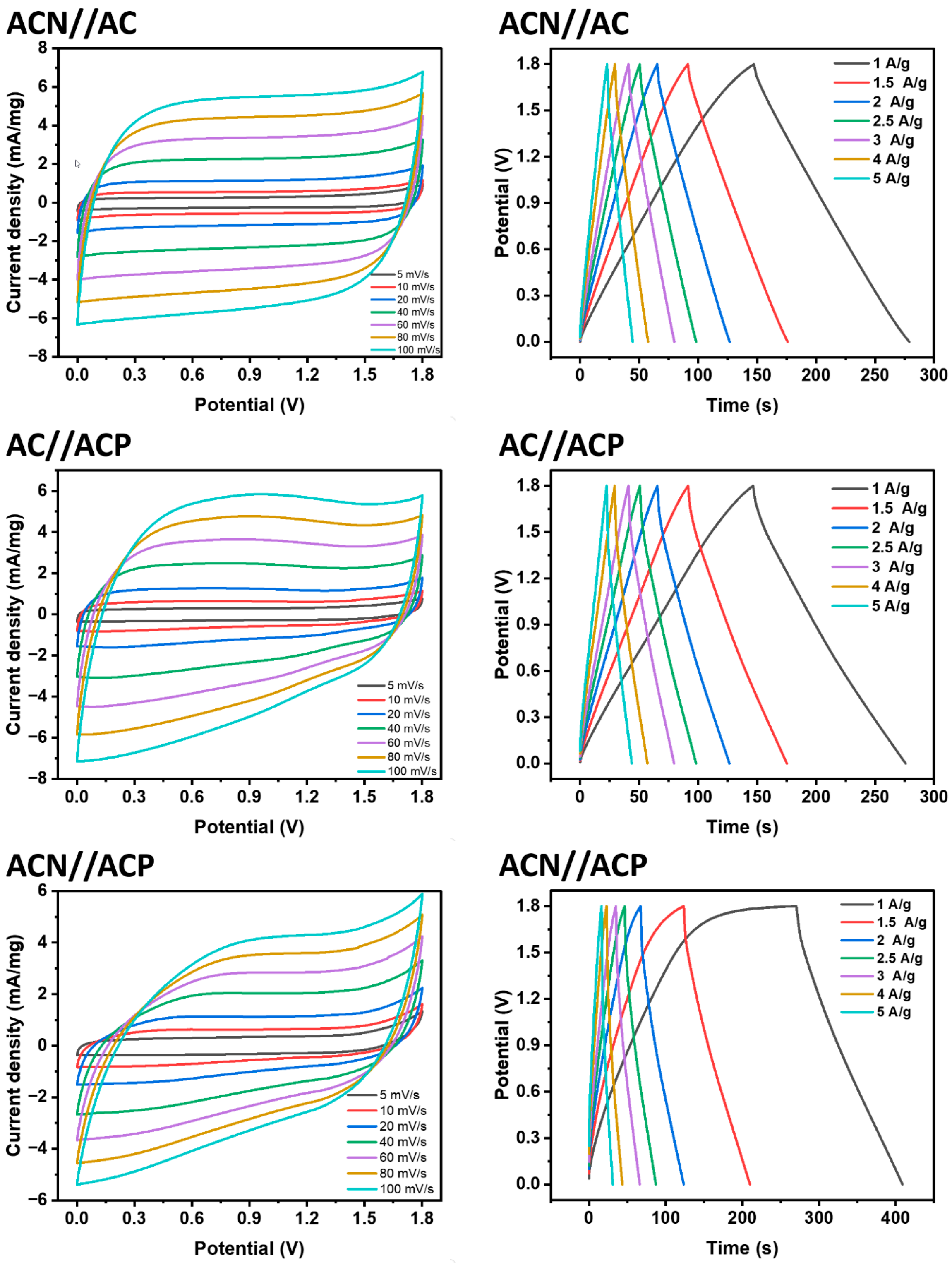

Figure S4. CV curves and Charge-discharge plots of asymmetrical **ACN//AC, AC//ACP**, and **ACN//ACP** cells.

Table S1. Specific capacitance calculated from the three-electrode system's GCD according to the current densities.

Current density (A/g)	Specific Capacitance (F/g)			
	AC-Cathode	ACP	AC-Anode	ACN
1	121.15659	173.70045	131.51735	150.21393
1.5	116.88222	172.35815	129.06968	147.49752
2	114.90402	166.4432	128.00957	143.34735
2.5	111.91934	164.10549	125.69255	139.90532
3	110.33573	160.44878	124.40431	136.80579
4	105.96947	157.0945	120.35656	132.14677
5	102.68126	152.89726	117.07006	127.10775

Table S2. Specific capacitance calculated from the symmetrical cells' GCD according to the current densities.

Current density (A/g)	Specific Capacitance (F/g)		
	AC	ACP	ACN
1	114.14407	150.80302	138.11989
1.5	113.01484	150.38368	137.36841
2	112.62564	145.88638	134.03188
2.5	110.49645	144.01715	130.87037
3	109.43526	140.4379	127.95139
4	105.71291	136.65754	123.39096
5	102.76055	132.20113	118.51297

Table S3. Specific capacitance calculated from the asymmetrical cells' GCD according to the current densities.

Current density (A/g)	Specific Capacitance (F/g)		
	ACN//AC	AC//ACP	ACN//ACP
1	36.58849	35.93071	38.46386
1.5	35.26439	34.90064	35.94422
2	34.09725	33.92003	31.21283
2.5	33.20054	33.07346	28.1991
3	32.36467	32.20717	25.88541
4	31.13266	30.80043	22.80141
5	30.04482	29.52675	20.38909
